# Nitrogen balance dynamics during 2000-2010 in the Yangtze River Basin croplands, with special reference to the relative contributions of cropland area and synthetic fertilizer N application rate changes

**DOI:** 10.1371/journal.pone.0180613

**Published:** 2017-07-05

**Authors:** Lijuan Wang, Hua Zheng, He Zhao, Brian E. Robinson

**Affiliations:** 1 State Key Laboratory of Urban and regional Ecology, Research Center for Eco-Environmental Sciences, University of Chinese Academy of Sciences, Beijing, China; 2 University of Chinese Academy of Sciences, Beijing, China; 3 Department of Geography, McGill University, Montreal, Québec, Canada; Universiteit Utrecht, NETHERLANDS

## Abstract

With the increases of cropland area and fertilizer nitrogen (N) application rate, general N balance characteristics in regional agroecosystems have been widely documented. However, few studies have quantitatively analyzed the drivers of spatial changes in the N budget. We constructed a mass balance model of the N budget at the soil surface using a database of county-level agricultural statistics to analyze N input, output, and proportional contribution of various factors to the overall N input changes in croplands during 2000–2010 in the Yangtze River Basin, the largest basin and the main agricultural production region in China. Over the period investigated, N input increased by 9%. Of this 87% was from fertilizer N input. In the upper and middle reaches of the basin, the increased synthetic fertilizer N application rate accounted for 84% and 76% of the N input increase, respectively, mainly due to increased N input in the cropland that previously had low synthetic fertilizer N application rate. In lower reaches of the basin, mainly due to urbanization, the decrease in cropland area and synthetic fertilizer N application rate nearly equally contributed to decreases in N input. Quantifying spatial N inputs can provide critical managerial information needed to optimize synthetic fertilizer N application rate and monitor the impacts of urbanization on agricultural production, helping to decrease agricultural environment risk and maintain sustainable agricultural production in different areas.

## Introduction

Nitrogen (N) is one of a number of critical plant nutrients that determine crop yield alongside, for example, phosphorus, potassium and a wide range of micro nutrients. The increase in consumption of world synthetic N fertilizers from10 Tg (1 Tg = 10^12^ g) N/y in the late 1950s to 100 Tg N/y in 2008 has played an important role in the rising rate of food production, as the global population and demand for food has increased [1]. Although the benefits of adding fertilizer N to agroecosystems are evident, costs arise in part because most of the N added to agroecosystems does not reach its ultimate aim—protein in the human diet. Only about 16% of N added as fertilizers is consumed by people [2,3], with some of the surplus N lost from the agroecosystem and potentially substantially altering downwind and downstream ecosystems [4]. Some transfers of reactive N in the form of a solution from terrestrial ecosystems (often agroecosystems) to streams, rivers, and ultimately the ocean, which will drive hypoxic zones and algal blooms and have other effects on the coastal ocean, can cause environmental problems (e.g., loss of biodiversity and eutrophication) [5] and alter the dynamics of coastal ocean systems [6]. Moreover, some portion of the soluble N leaches deep into groundwater, finally affecting human health [7]. Some transfers of reactive N are in the form of gas into the atmosphere; for example, the nitrogen oxides (NOx) formed from ammonia volatilization (NH_3_) and denitrification under the influence of thermal infrared radiation and ozone, contribute to the greenhouse effect and acid rain [8], and ultimately affect human health [7].

Given the serious environmental problems caused by adding fertilizer N and the balance between supply and demand for N, agricultural N management is vital for ensuring sustainable agricultural practices and environmental protection. The central target of agricultural N management is to enhance agricultural productivity to reduce famine, feed a growing population, and support the increasing demand for food, while simultaneously reducing the transfer of reactive N to non-target ecosystems. It is therefore important to understand the cycle of N in cropland, especially the inputs (e.g. synthetic fertilizers, biological N fixation, and recycled N and atmospheric deposition), outputs (e.g. N in harvested crops, denitrification, NH_3_ volatilization and N transported to water bodies through run-off and leaching), pathways of mobilized or retained reactive N [9], and N use efficiency (NUE: the ratio of N input and yield output of the system) [10].

In many countries, N budgets have been estimated, for different periods at various scales, including by county, watershed, region, and worldwide[11–13]. Similarly, many studies have addressed different aspects and scales of the N budget issue in China, such as the Yangtze River, Haihe, and Taihu Basin [14,15]. Several studies have addressed different periods of the N budget associated with the Yangtze River Basin. Studies have estimated N biogeochemical cycling in the Yangtze River Drainage Basin[16], estimated N budgets of agricultural fields in the Yangtze River Basin from 1980–1990 at the county level [17] and focused on the N budget and its changes in 1980, 1990 and 2000 using a mass balance model over the whole basin [18]. How socio-economic factors influence N consumption and surplus was also explored [3,19,20]. However, few studies have quantified the relative contributions of cropland area and fertilizer N application rate changes for the fertilizer N input at the watershed scale, and further explained the factors responsible for these spatial changes in upper, middle and lower reaches. Quantifying the fertilizer N input change due to the changes of cropland area and fertilizer N application rate will help provide critical managerial information for decreasing watershed environmental risk and maintaining sustainable agricultural production in different areas.

We examined the N inputs, outputs and their changes during 2000–2010, based on a mass balance model using updated agricultural statistics of 856 counties in the Yangtze River Basin. Using available literature from the region, we determined the N surplus intensity (divided the N balance value of the basin by its area of cropland) and spatio-temporal changes of NUE using a geographical information system(GIS) model, which allowed us to manage various data in a common spatial framework. Then we assessed the influence and drivers of the spatio-temporal changes in the N balance. Our aim was to determine how changes in N balance and fertilizer N might be influenced by land-use changes and fertilizer N application rate and then provide explicit managerial implications of agricultural N fertilizer for different parts of the Yangtze River Basin.

## Materials and methods

### Study areas

**Fig 1:**
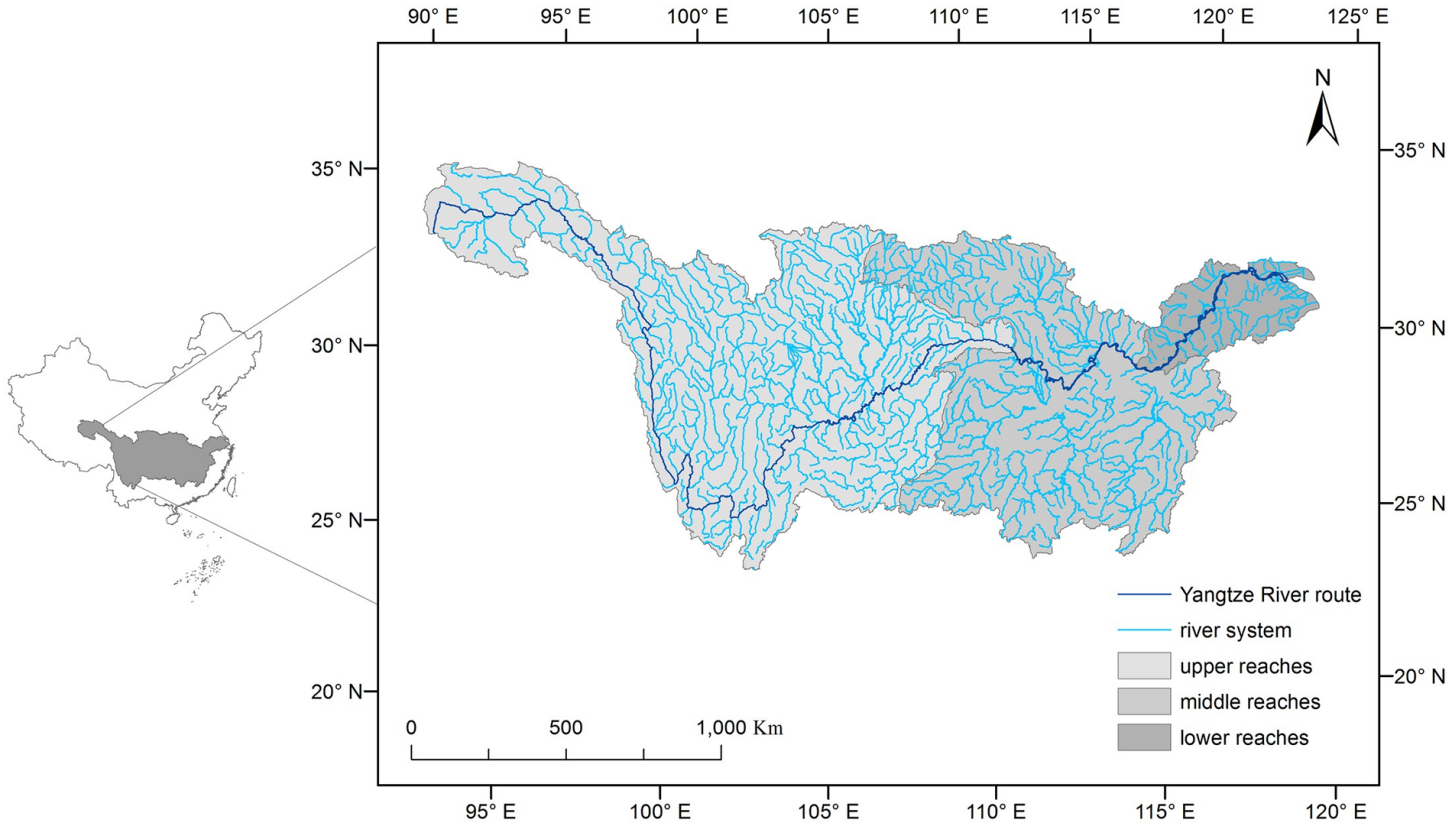
Location of the Yangtze River Basin in China.

The Yangtze River Basin is located in the middle of southern China (Fig 1). The Yangtze River is the largest river in China and the third largest in the world. Its mainstream length is 6380 km, total area is more than 1.81 × 10^6^ km^2^ and average annual discharge into the East China Sea is 9.79 × 10^11^ m^3^ [18]. The tributaries extend to eight provinces (autonomous regions). In China, the Yangtze has long been considered in three parts(upper, middle and lower reaches), based on differences in geological conditions and hydrological systems [18]. The dividing point of the upper reaches of the river is Yichang in Hubei Province,. the middle reaches extend from Yichang to Hukou and the lower reaches are below Hukou (Fig 1).

The Yangtze River Basin is one of the most densely populated and agriculturally productive areas in China. It accounts for 19% of China's land area [16]. In 2010, 450 million people lived in the Yangtze River Basin, representing 34% of China's population. Its grain yield accounts for about 37% of the total national crop production and 70%-75% of the national rice harvest [18]. With the rapid increment of economy and population, some areas of the basin are experiencing fast urbanization and industrialization [21,22]. Additionally, the basin also faces environmental degradation to the aquatic environment, risk of flood disaster and eutrophication [23]. Most of the eutrophic lakes occur in the middle and lower reaches of the Yangtze River [24].

### Calculating N balance

Because major sources or sinks are difficult to measure independently, a mass balance model was used to estimate N input and output in the croplands [25] of the Yangtze River Basin, which were the focus of this study. The N inputs include synthetic fertilizer N, biological N fixation, recycled N, and atmospheric deposition N [18,26]. The N outputs include N in harvested crops, denitrification, NH_3_ volatilization, and riverine N loss by leaching and run-off [17,18]. The N balance value was estimated from the difference between all inputs N and crop harvest N. We divided the N balance value of a county by its area of cropland to calculate the N surplus intensity (NSI) [15]. We divided the proportion of all N inputs by N in the harvested crops to obtain the NUE [13]. The N budget was calculated for the years 2000 and 2010 for each county of the Yangtze River Basin. The aim of our research was to calculate the inputs and outputs of N, and changes in the N balance characteristics, as well as determine the spatial distribution of NUE and NSI and defining the drivers of N inputs. This would allow us to determine how changes in N balance and fertilizer N might be influenced by land-use changes and fertilizer N application rate and so provide managerial implications of agricultural N fertilizer in the watershed.

### Calculating N input

The N inputs include synthetic fertilizer N(N_chemin_), biological N fixation (N_fin_), recycled N (N_recycn_) and atmospheric deposition N (N_atmin_). Recycled N includes animal excrement N (N_livestin_), rural human excrement N (N_human_) and crop residues used as N fertilizer (N_strawin_).

$$N_{input}=N_{che\text{min}}+N_{at\text{min}}+N_{fin}+N_{recycn}$$

$$N_{recycn}=N_{human}+N_{livestin}+N_{strawin}$$

1. N_atmin_
  The N_atmin_ is the result of both natural and anthropogenic reactive N emissions, which includes wet and dry deposition in the form of inorganic and organic N [15]. However, only the bulk deposition of ammonium (NH_4_^+^) N and (NO_3_^-^) N are available because this is more easily measured [27]. We calculated bulk NH4^+^-N and NO_3_^—^N deposition by multiplying the agricultural land area (A) and the unit area amount of bulk NH_4_^+^-N and NO_3_^—^N deposition (P), using the unit area amount of bulk NH4^+^-N and NO_3_^—^N deposition rate from a recent study [28]. The data were based on 671 data points concerning the bulk deposition of NH4^+^-N and NO_3_^—^N in terrestrial ecosystems. We use different bulk depositions of N in the upper, middle and lower reaches of the basin in different years. In Qinghai and Xizang Province, we used 3.65 kg N/ha/y in 2000 and 5.31 kg N/ha/y in 2010 as the deposition rate. In other upper and middle reaches, we used 17.97 kg N/ha/y and 23.28 kg N/ha/y in 2000 and 2010 respectively, and corresponding in lower reaches, we used 20.95 and 26.55 kg N/ha/y.
  $$N_{at\text{min}}=AP$$
2. N_chemin_
  In our study, N_chemin_ consists of pure N fertilizer (N_nitrogen_) and N in compound fertilizer (N_compin_). According to the data of the China Agriculture Yearbook for 2009[29], the ratio of N, phosphorus (as P_2_O_5_) and potassium (as K_2_O) of compound fertilizer differed for different areas. In the Yangtze River Basin, the N: P: K ratio was 1:1:0.8. Therefore, we used 35% as the average N content of compound fertilizer [30,31].
  $$N_{che\text{min}}=N_{nitrogen}+0.35N_{compin}$$
3. N_fin_
  The growing of legumes was the main source of N_fin._ The N inputs from N_fin_ include contributions from both symbiotic N-fixation crops (peanut and soybean) and non-symbiotic N-fixation crops (wheat and rice) [32]. The N inputs from biological fixation were equal to the product of the average N-fixation rates of each crop (F_i_) and the sown area (A_i_) [17,33,34]. According to previous studies of N-fixation rates in China, we used average N-fixation rates of 80 kg N/ha/y for peanut [35] and soybean, 30 kg N/ha/y for rice and 15 kg N/ha/y for wheat [30,34].
  $$N_{fin}=\overset{n}{\underset{i=1}{\sum }}A_{i}F_{i}$$
4. N_recycn_
  Harvested crops are consumed by animals (e.g. cattle, pigs and sheep) and humans, and crop residues (straw) are used as fertilizer and for burning. In China, human and animal excrement was traditionally used as organic manure for cropland. Therefore, the annual recycled N input was the sum of the animal excrement N (N_livestin_), rural human excrement N (N_human_), crop residue N used as fertilizer (N_strawin_). our parameters were based on the study in the Yangtze River basin [18].
  1. Animal excrement and rural human N
    The annual N_livestin_ was equal to the product of the animal population quantity (P_i_) and the unit N emission coefficient per animal per year (E_i_).The unit N emission coefficients for pigs, cattle and sheep were 8, 40 and 5 kg N/ha/y, respectively [16,17,33,34,36].
    The annual N_human_ was equal to the product of the rural population (P) and the unit N emission coefficient per person per year (E). We calculated the rural total rural population from the rural adult population using a conversion coefficient of 0.85 and the unit N emission coefficient for a human was 5 kg N/ha/y [30]. The input from people and livestock excrement N applied to farmland as manure accounted for 40% of the total N input [17]. The remaining of livestock and human excrement N could be discharged into waterbodies, emitted by volatilization and denitrification, and left unused [37,38]
    $$N_{human}=0.85\times P\times E\times 0.4$$
    $$N_{livestin}=\sum_{i=1}^{n}0.4P_{i}E_{i}$$
  2. Crop residue N used as fertilizer
    Many kinds of crops residues are used as fertilizer for soil in the Yangtze River Basin, however, data on crop residues are not available. Therefore, we estimated the crop residue N used as fertilizer (N_strawin_) using the straw/seed ratio (Ri), the percentage of residues returned to the soil (S_i_), the annual yield of crop (Y_i_) and the N concentration in crop residues(P_i_) [17,39]. Our parameters were based on a study in the Yangtze River Basin [18].
    $$N_{strawin}=\sum_{i=1}^{n}Y_{i}R_{i}S_{i}P_{i}$$

### Calculating N output

The N_output_ includes N in harvested crops(N_harvout_) and other loss pathways to the environment crop residues (straw) are burned (N_burn_), denitrification, and NH_3_ volatilization and N transported to water bodies and stored in cropland).

1. N_harvout_
  The N_harvout_ was calculated by conversion from protein. We calculated the amount of N in the harvested crops from the yields of major crops and data on N concentrations in crops [30], using parameters based on a previous study [18].
2. Denitrification and NH_3_ volatilization
  Many factors influence the emissions from denitrification and volatilization, such as tillage practices and type and rate of fertilizer. Previous experiments in the basin calculated that denitrification and volatilization originated mainly from fertilizers and human and animal wastes. Denitrification includes losses from paddy fields 36%, uplands 25%, and organic manure15% [18].
  The N losses in the agriculture from fertilizer and human and animal waste are mainly by NH_3_ volatilization. We omitted the N_2_O and NO emission because they represent a very small proportion of all inputs [38]. The NH_3_ volatilization includes losses from paddy fields 16%, uplands 11%, and organic manure 23%[18,40].
3. Riverine N loss by leaching and run-off
  It is complicated to measure riverine N loss by leaching and run-off because of the numerous influence factors, such as land use type, soil type, drainage network and reservoirs. We use the average loss rate from the literatures to calculate surface run-off and leaching N. The average rates of run-off loss of chemical N applied to croplands for paddy fields and uplands were 5.2 and 11%, respectively [38]. We used averaged loss rates of 1.2 and 3.2% as the leaching loss from chemical fertilizer applied to paddy fields and uplands, respectively. All organic N discharged to water bodies as run-off and leaching were 5 and 4%, respectively [38].
4. Crop residue combustion N
  We calculated the N from crop residue combustion based R_i_, Y_i_ and the percentage of residues burned for fuel (B_i_) and the conversion coefficient for estimating the NOx, N_2_O,N_2_ and NH_3_ formed from burning crop residues (C_i_). The conversion coefficient is equal to 3.83g/kg [17,39,41].
  $$N_{burn}=\sum_{i=1}^{n}Y_{i}R_{i}B_{i}C_{i}$$

### Proportional contribution of cropland area and fertilizer N application rate to fertilizer N input

The development of a simple attribution approach is similar to that taken in studies that have used the Kaya identity to obtain the regional drivers of accelerating carbon dioxide emissions [42], and forest identity [43]. Therefore, we used fertilizer N identity to determine the relative contribution of changes in cropland area and fertilizer N application rate in different parts of the Yangtze River Basin. We assessed the relative contribution of changes in cropland area and fertilizer N application rate to the change in total fertilizer N input using Eqs (1) and (2).

M=A×D (1)

Because ln(*M*) = ln(*A*) + ln(*D*)

The rates of change (m, a, and d) of M, A, and D are:

$$m\approx \frac{d\text{ln}\left(M\right)}{dt},a\approx \frac{d\text{ln}\left(A\right)}{dt},d\approx \frac{d\text{ln}\left(D\right)}{dt},$$

Then,

m=a×d (2)

where, M, A and D represent the total fertilizer N input (Tg N), cropland area (ha) and fertilizer N application rate (kg N/ha), respectively; and m, a, and d are the corresponding derivatives (or rates of change) of these attributes over time. This identity combines the values of cropland area with the fertilizer N application rate to give the change in fertilizer N input.

Regression analysis was conducted between the socio-economic factors (rural population and urban population density, grain yields (rice, wheat, peanuts and soybean), the fertilizer N application rate in 2000 and the changes of fertilizer N application rate and cropland area in the upper, middle and lower reaches of the Yangtze River Basin. This was used to reveal the factors driving fertilizer N input changes through changing cropland area and fertilizer N application rate. All analyses were conducted using the software Stata (version 14.0; Stata MP, College Station, TX, USA).

### Data sources

We created a county-level database using GIS technology. First, we used county-level agricultural statistical data for 2000 and 2010 (from the Chinese Academy of Agricultural Sciences) to calculate the N budgets of the Yangtze River Basin, including fertilizer type and amount, crop type (e.g. wheat, corn and rice) and yields, cropland area, paddy land area, upland area, and livestock type (e.g. pigs, sheep and cattle) and amount. The crop area was obtained from the Chinese planting industry information network. Then, we used GIS software to analyze the spatial data (provided by the Institute of Remote Sensing and Digital Earth Chinese Academy, Beijing, China), including the county boundary data for countries and the Yangtze River Basin.

### Uncertainty analyses of N budgets

Because of the uncertainty arising from the use of literature values and extrapolating these to the Yangtze River Basin area, we performed a sensitivity analysis by Monte Carlo simulations in Crystal Ball 7.0 software (Decisioneering, Inc., Denver, CO, USA) run 20000 times in 2000 to illustrate the importance of the assumptions for the results of this budgeting exercise.

The Chinese statistical data were collected using a new system during 2000 and 2010, and we believe that the county-level agricultural statistical data were an authentic source for our analyses[38]. We used different bulk depositions of N in the upper, middle and lower reaches of the Yangtze River Basin in different years, and we believe these data were also credible [28]. Therefore, the uncertainties were mainly from the emission rates and factors. The Monte Carlo simulations can estimate the contribution of the factors to the budget by characterizing their statistical distribution functions. For N content of compound fertilizer, a coefficient of variation (CV) of 5% was assumed, for N fixation, all CVs were assumed to be 35%, and for human and livestock emission factor and rate, CVs of 30 and 20% were used, respectively [31].

## Results

### N inputs, outputs and changes

The total N inputs of the Yangtze cropland increased by 1.2 Tg during 2000-2010. The largest contributor to the increased N input was fertilizer N, which also accounted for 87% of the total N input changes over the ten years. The N in the harvested crops increased by 0.6 Tg. The total N budget of the Yangtze cropland increased by 0.6 Tg. The N loss through NH_3_ volatilization, denitrification and N transported to water bodies increased slightly decreased slightly (Table 1).

**Table 1:** Estimated agricultural N inputs and outputs (Tg) in the Yangtze River Basin for 2000 and 2010.

	2000	2010
**Fertilizer N**	**8.4**	**9.5**
**Biological N fixation**	**1**	**0.9**
Symbiotic N-fixation	0.4	0.2
Non-symbiotic N-fixation	0.6	0.6
**Atmospheric deposition**	**0.5**	**0.6**
**Recycled N**	**2.9**	**3.1**
Crop residue used as fertilizer	0.9	0.9
Animal and human excrement	2.1	2.2
**Total inputs**	**12.8**	**14**
**N in the harvested crops**	**4.6**	**5.2**
Wheat	0.5	0.6
Maize	0.6	0.7
Rice	1.7	1.8
Soybean	0.4	0.4
Vegetables	0.7	0.8
Fruits	0.04	0.1
Oil crops	0.5	0.6
Other crops	0.2	0.2
**Budget (inputs-crop harvest)**	**8.3**	**8.9**
**Crop residue combustion**	**0.5**	**0.6**
**NH**_**3**_ **volatilization**	**1.8**	**2**
Paddy rice	0.7	0.8
uplands	0.4	0.5
Organic manure	0.7	0.7
**Denitrification**	**3.1**	**3.4**
Paddy rice	1.7	1.7
Uplands	1	1.2
Organic manure	0.4	0.5
**N transported to water bodies**	**1.1**	**1.3**
**N stored in cropland**	**1.7**	**1.7**

### Spatial changes in NSI and NUE

**Fig 2:**
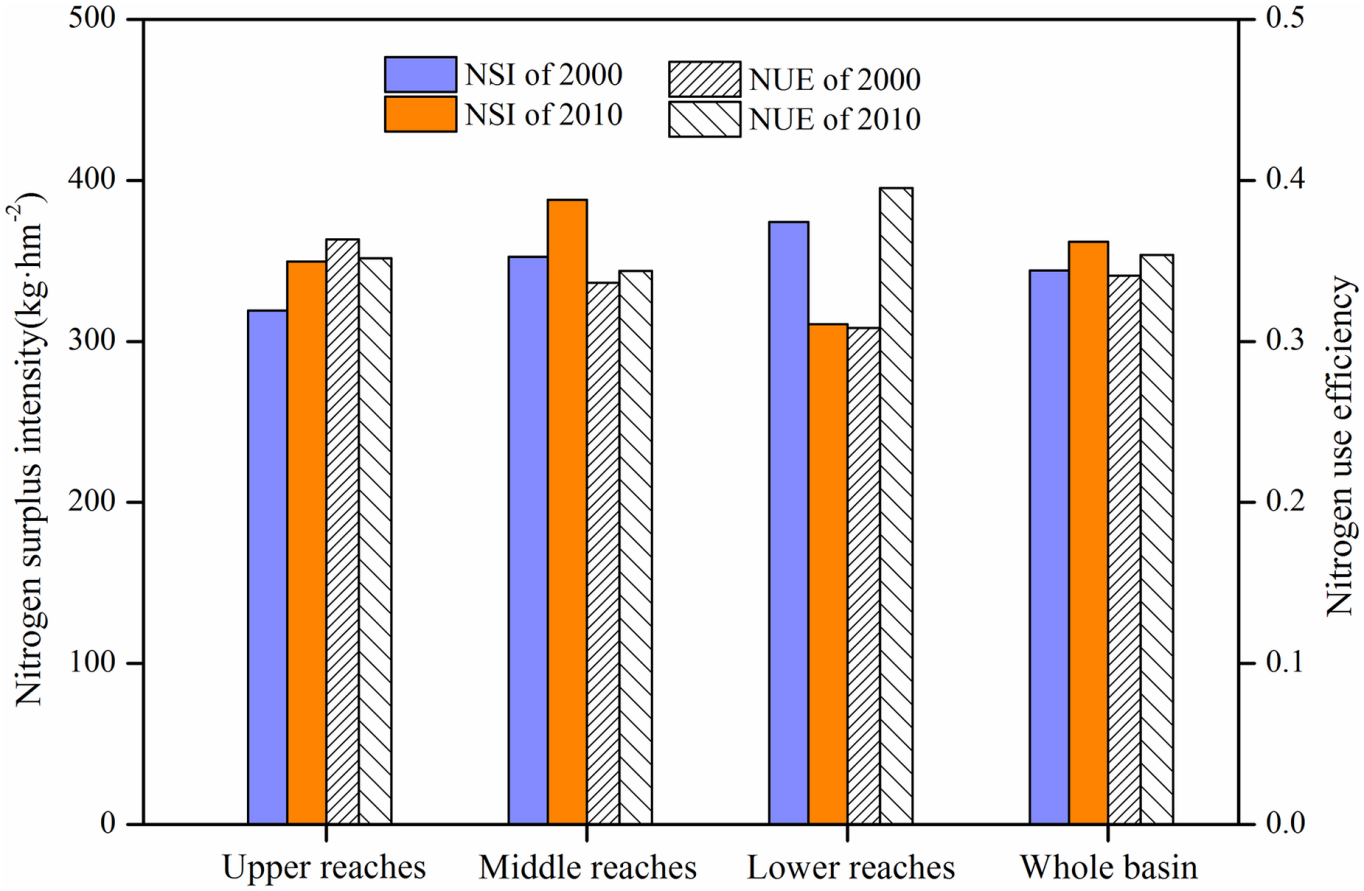
Changes in NSI and NUE in the upper, middle and lower reaches of the Yangtze River Basin.

**Fig 3:**
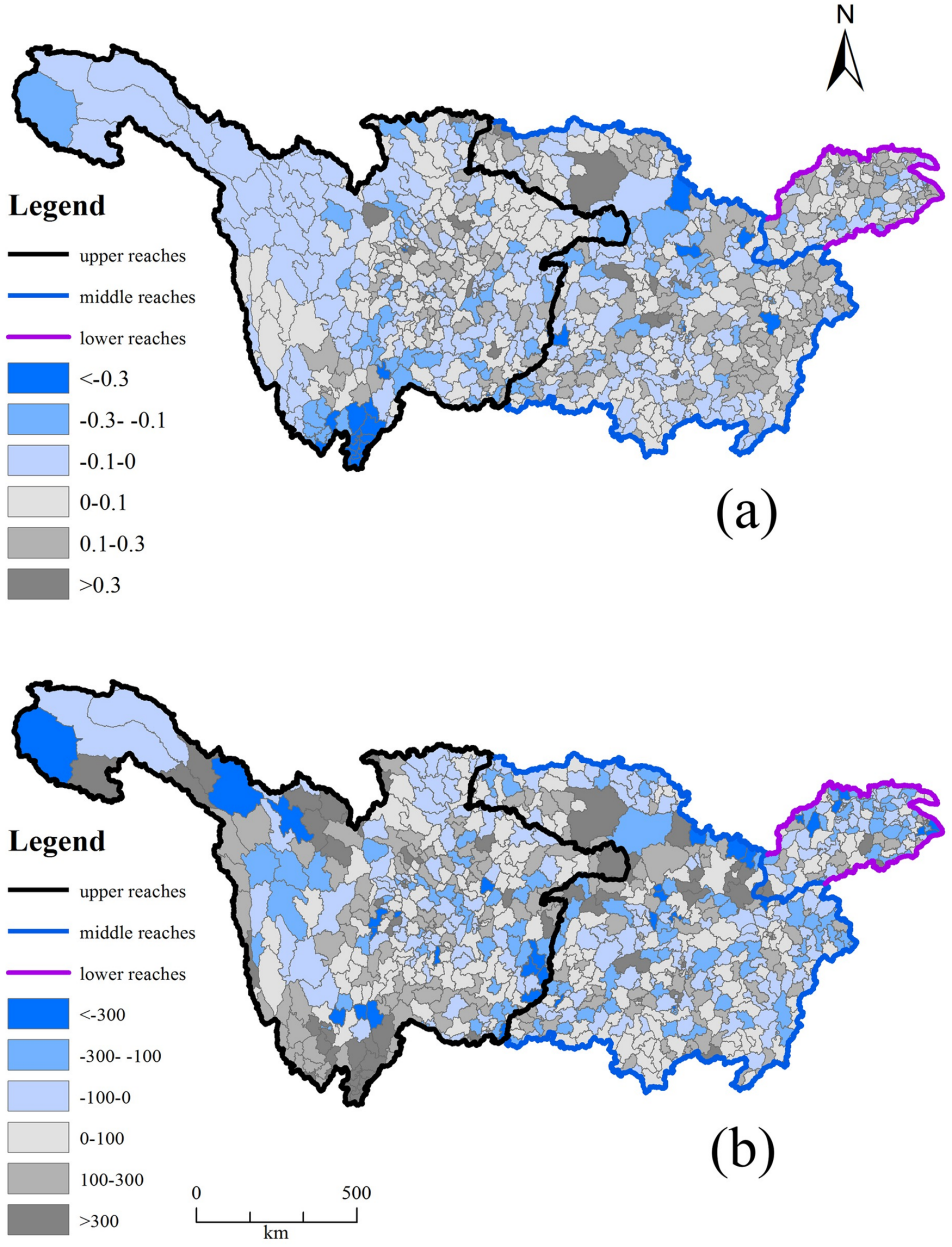
Spatial changes of NSI (a) and NUE (b) during 2000–2010 in the Yangtze River Basin.

There were spatial variations in NSI changes. The maximum increase (35kg/ha) in the NSI occurred in the middle reaches followed by the upper reaches of the basin (30 kg/ha). However, NSI of the lower reaches decreased by 60 kg/ha (Figs 2 and 3).

The NUE of the Yangtze River Basin cropland changed slightly, and with spatial variations in the changes. The NUE decreased slightly for the upper reaches, did not change for the middle reaches and increased by 0.1 in the lower reaches (Figs 2 and 3).

### Relative contributions of cropland area and fertilizer N application rate to fertilizer N input

**Fig 4:**
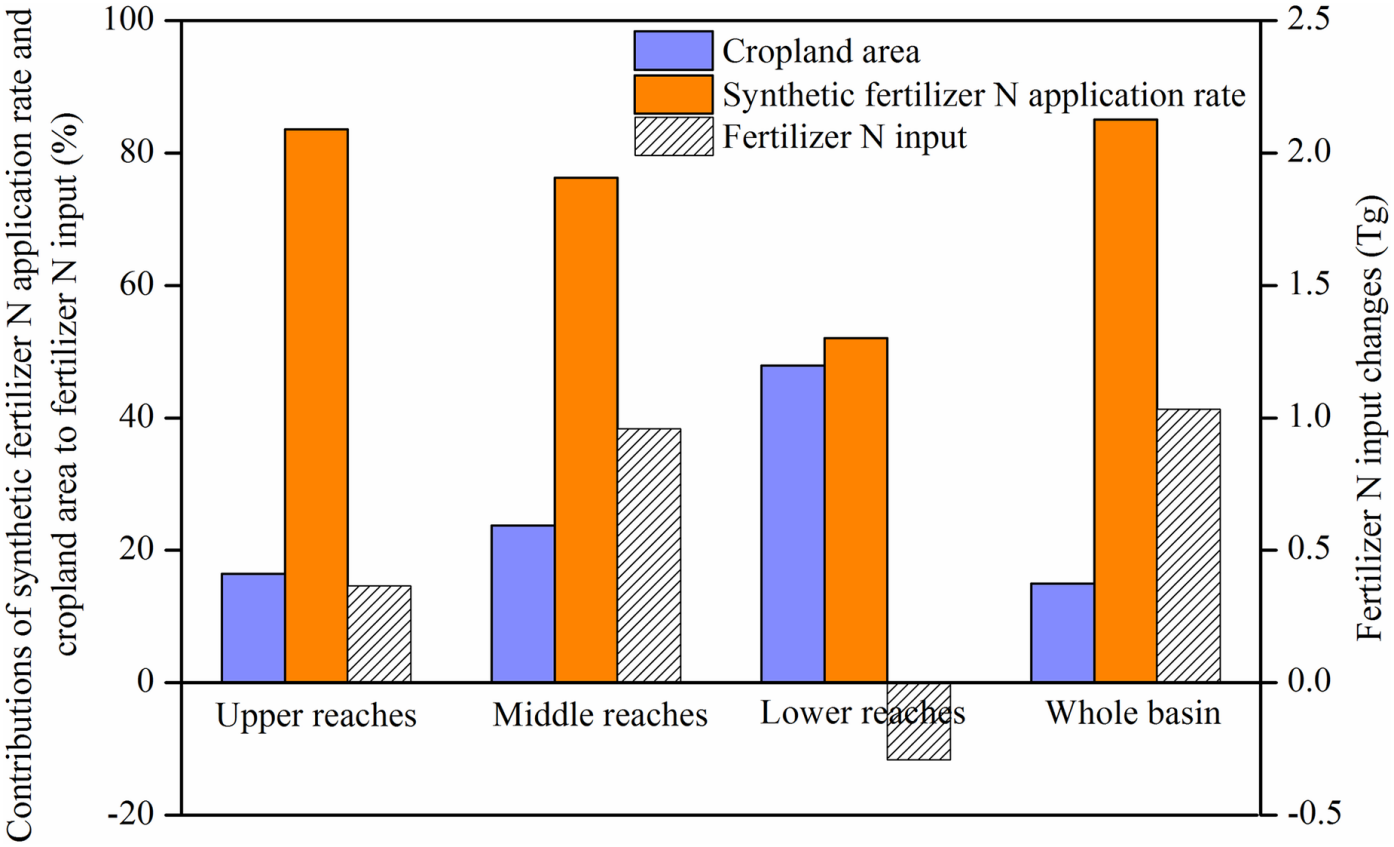
Relative contributions of cropland area and synthetic fertilizer N application rate to fertilizer N input during 2000–2010.

The increase in the N fertilizer input was mainly affected by the area of cropland and the fertilizer N application rate. The area of cropland area increased by 0.2%, fertilizer N application rate increased by 10%, and the fertilizer N input increased by 1.1 Tg from 2000 to 2010. The fertilizer N input in the upper and middle reaches of the Yangtze River Basin increased, but it decreased in the lower reaches (Fig 4).

The extent of the contributions of the area of cropland and the synthetic fertilizer N application rate to the change in fertilizer N input differed. The area of cultivated land and the contributions of the synthetic fertilizer N application rate in the whole of the Yangtze River Basin were 15% and 85%, respectively. Similarly, in the upper and middle reaches, the increase in the synthetic fertilizer N application rate was the main factor responsible for the increased N fertilizer input (representing 84% and 76% of the total N input, respectively). However, the decreased N fertilizer input in the lower reaches was due to both the area of cropland and the decrease of synthetic fertilizer N application rate, with proportional contributions of 48 and 52%, respectively (Fig 4).

In the upper and middle reaches, increased synthetic fertilizer N application rate mainly resulted from croplands with low fertilizer N application rate in 2000. For the increase of fertilizer N application rate in upper and middle reaches, coefficients for fertilizer N application rate in 2000 were negative and significant (P < 0.01 and P < 0.05, respectively) (Table 2). In the lower reaches, urbanization contributed significantly to the decrease in fertilizer N application rate and cropland area. For fertilizer N application rate and cropland area, coefficients for proportion of construction land area in 2000 were negative and significant (P < 0.05 and P < 0.001, respectively), however, coefficients for change rate of construction land area were positive (P < 0.01 and P < 0.001, respectively) (Table 2).

**Table 2:** Factors associated with the change of synthetic fertilizer N application rate and cropland area in upper, middle and lower reaches of the Yangtze River Basin.

Category	Independent variable	Increase of fertilizer N application rate in upper reaches	Increase of fertilizer N application rate in middle reaches	Decrease of fertilizer N application rate in lower reaches	Decrease of cropland area in lower reaches
Initial condition	Fertilizer N application rate in 2000 (kg/ha)	-0.212** (7.80E-04)	-0.338* (0.002)	0.509** (1.22E-04)	——
	Proportion of cropland area in 2000 (%)	——	——	——	0.291 (0.109)
Crop production	Rice yield in 2000 (kg/ha)	0.074 (1.53E-04)	-0.039† (2.71E-05)	-0.234 (0.022)	-0.174 (0.011)
	Change rate of rice yield 2000–2010 (%) ^a^	0.013 (0.103)	-0.175 (0.223)	-0.400 (0.077)	-0.291† (0.037)
	Wheat yield in 2000 (kg/ha)	-0.049 (0.041)	0.808 (0.111)	0.117 (6.20E-05)	-0.052 (2.26E-05)
	Change rate of wheat yield 2000–2010 (%)	-0.113*** (0.062)	0.743 (0.094)	0.115 (0.038)	0.026 (0.007)
	Corn yield in 2000 (kg/ha)	——	0.049 (0.059)	0.132 (2.14E-04)	——
	Change rate of corn yield 2000–2010 (%)	0.358*** (0.055)	-0.037 (0.019)	-0.198 (0.003)	-0.029 (0.012)
	Soybean yield in 2000 (kg/ha)	0.020 (0.243)	-0.073 (0.669)	——	-0.146 (4.44E-05)
	Change rate in corn yield 2000–2010 (%)	-0.013 (0.034)	-0.303 (0.435)	0.259 (0.065)	-0.069 (0.015)
	Peanut yield in 2000 (kg/ha)	0.074 (0.485)	0.019 (0.380)	——	——
	Change rate in peanut yield (%)	-0.034† (0.006)	0.121 (0.076)	-0.388† (0.045)	0.035 (0.009)
Urbanization	Rural population density in 2000 (capita/km^2^)	0.119* (0.024)	0.122 (0.049)	0.125 (0.008)	0.564* (0.004)
	Change rate in rural population density 2000–2010 (%)	0.754*** (0.053)	0.046 (0.129)	-0.010 (0.228)	0.453** (0.084)
	Urban population density in 2000 (capita/km^2^)	-0.093 (4.50E-04)	-0.018 (2.58E-05)	0.236† (1.70E-04)	-0.019 (6.10E-05)
	Change rate of urban population density 2000–2010 (%)	0.062*** (0.001)	-0.075 (0.028)	0.384 (0.071)	-0.007** (0.032)
	Proportion of construction land area in 2000 (%)	0.050 (5.590)	0.025 (6.893)	-0.681* (0.613)	-0.705*** (0.257)
	Change rate of construction land area 2000–2010 (%)	-0.026 (3.766)	0.018 (15.329)	0.979** (0.885)	0.789*** (0.271)
R^2^	——	0.704	0.385	0.657	0.497
N	——	177	139	39	57

Unit of analysis is the county. Dependent variables are increases in per-unit-area carbon sequestration, soil retention, and water retention, respectively. Standardized coefficients and robust standard errors are reported outside and inside parentheses, respectively. Model results passed standard regression diagnostics. Variance inflation factors were tested to be <10.

^†^, P < 0.1;

*,P < 0.05;

**, P < 0.01;

***,P < 0.001.

^a^Change rate during 2000–2010 = [(amount in 2010 –the amount in 2000) / amount in 2000] × 100%.

### Uncertainty analyses of N budgets

**Fig 5:**
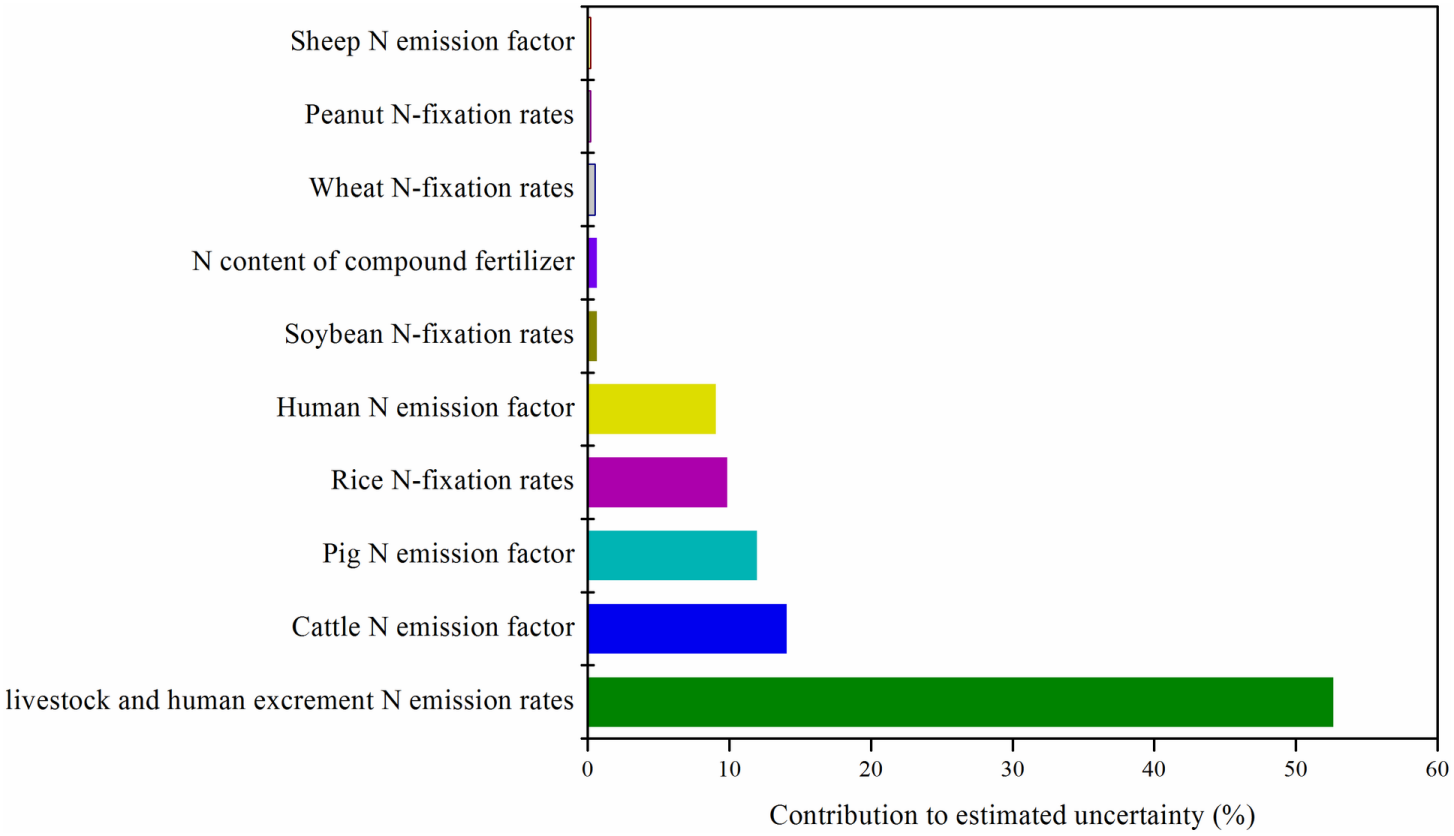
Contributions of various parameters to variability of N budget of 2000.

After running 20000 Monte Carlo simulations, the average N budget of 2000 was 8.1 Tg with a 90% uncertainty range of 6.3–11.4Tg. The largest variance stemmed from livestock and human excrement N emission rates, which accounted for 52% of the variability of the N budget. Cattle and pig N emission factor accounted for 14 and 12%, respectively (Fig 5).

## Discussion

There were clear spatial and temporal variations in the Yangtze River Basin. The changes of NSI and fertilizer N input during 2000–2010 differed but their spatial changes were similar. The NSI and fertilizer N input changes during 2000–2010 in the upper and middle reaches of the basin increased, but it decreased in the lower reaches. Fertilizer N is usually the largest source of total N input [20,44–46] and account for 87% of total N input in this study. The increase of NSI was usually related to the increase in fertilizer N input. The results were similar to those of previous studies with about 71% of the N input coming from fertilizer N in croplands and causing many environmental problems by entering waterbodies or the atmosphere, destroying the balance of ecosystems and harming human health [2,45].

We further identified the relative contributions of changes in cropland area and fertilizer N application rate to fertilizer N input changes in the different parts of the Yangtze River Basin. Fertilizer N application rate was the main factor resulting in increased fertilizer N input in the upper and middle reaches, fertilizer input decreased in the lower reaches due to the decrease of fertilizer N application rate and cropland area (Fig 4). These results were similar to a study of N inputs for the Yangtze River Basin and suggested that the intensity of agricultural activities increased gradually from the lower to the middle and upper reaches mainly through increases in synthetic fertilizer N application rate, and fertilizer N was the major input for the middle and lower reaches [26].

Based on the relative contributions of cropland area and synthetic fertilizer N application rate changes to fertilizer input (Fig 4), the main factors influencing the changes in fertilizer N application rate and cropland area were further analyzed. The increase of fertilizer application rate in croplands with low fertilizer N application rate in 2000 contributed to the increase of fertilizer N application rate in upper and middle reaches (Table 2). However, in lower reaches, the decrease of fertilizer N application rate and cropland area due to urbanization lead to the decrease of fertilizer N input (Table 2). These results were similar to the study in the lower reaches of Yangtze River Basin, For example, rapid urbanization and industrialization of Jiangsu Province (in the lower reaches) occupied rice paddy fields and dryland [21]

Quantitative analysis of the contributions to fertilizer N input can also provide important information for spatial management of risk decrease in the agricultural environment. In the upper and middle reaches, the focus should be on monitoring and optimization of fertilizer N application rate [47,48], especially in croplands that previously had low fertilizer N application rate during agricultural development. Moreover, recent study suggested that joint study of N and phosphorus is important to understand surplus and balance and improve their use efficiency [49]. It is also necessary to clearly understand the relationship between N inputs and possible N yield [48]. In the lower reaches, the focus should be on the impacts of urbanization on agricultural production. These conclusions are in accord with a study of net anthropogenic N inputs in the upper Yangtze River Basin, in which fertilizer N input decreased with increased urbanization in the city of Chengdu [50]. The strictest farmland protection system should be implemented to prevent the decline of arable land and basic farmland area, it plays an important role in farmland protection. In addition, spatial differences in crop composition and crop management strategies may also be responsible for spatial differences in fertilizer N input, NSI and NUE [20,47,51], and these should be analyzed further.

There were some uncertainties in calculation of the N budget, mainly caused by obtaining some parameters from the literature, such as N deposition and N from animal and human excrement. During this study, we tried to use approximate parameters (e.g., N contents in products and N deposition) from local data in different areas of the Yangtze River Basin, to make our results more valid and reliable. In addition, we did not consider grassland and wetland, which may influence the N budget of croplands. The results of uncertainty analyses of N budgets showed that the largest variance stemmed from livestock and human excrement N emission rates (Fig 5). Therefore, to reduce the influence of uncertainty in future study, the anthropogenic parameters for N budgets in rural regions should be identified [52].

We revealed the spatial heterogeneity of synthetic fertilizer N input changes during 2000–2010 and quantitatively analyzed the contribution of different factors to these changes in the Yangtze River Basin. In the upper and middle reaches, the increased synthetic fertilizer N application rate, mainly due to increased N input in cropland that previously had low fertilizer N application rate, accounted for 84% and 76% of the N input increase, respectively. However, in lower reaches, mainly due to urbanization, the decreased cropland area and fertilizer N application rate nearly equally contributed to the fertilizer N input decrease. The quantitative analysis of different factors contributing to fertilizer N input can provide spatial information needed to optimize synthetic fertilizer N application rate and monitor the impacts of urbanization on agricultural production [53], helping to decrease agricultural environment risk and maintain sustainable agricultural production in different areas.
